# Positron Spectroscopy of Nanodiamonds after Hydrogen Sorption

**DOI:** 10.3390/nano8010036

**Published:** 2018-01-11

**Authors:** Lyudmila Nikitina, Roman Laptev, Yuri Abzaev, Andrey Lider, Alexander Ivashutenko

**Affiliations:** 1 Department of General Physics, Institute of Physics and Technology, Tomsk Polytechnic University, 634000 Tomsk, Russia; laptevrs@tpu.ru (R.L.); lider@tpu.ru (A.L.); ivashutenko@tpu.ru (A.I.); 2 Department of Higher Mathematics, Faculty of General Education, Tomsk State University of Architecture and Building, 634003 Tomsk, Russia; Abzaev2010@yandex.ru

**Keywords:** nanodiamonds, positron spectroscopy, hydrogen sorption

## Abstract

The structure and defects of nanodiamonds influence the hydrogen sorption capacity. Positronium can be used as a sensor for detecting places with the most efficient capture of hydrogen atoms. Hydrogenation of carbon materials was performed from gas atmosphere. The concentration of hydrogen absorbed by the sample depends on the temperature and pressure. The concentration 1.2 wt % is achieved at the temperature of 243 K and the pressure of 0.6 MPa. The hydrogen saturation of nanodiamonds changes the positron lifetime. Increase of sorption cycle numbers effects the positron lifetime, as well as the parameters of the Doppler broadening of annihilation line. The electron-positron annihilation being a sensitive method, it allows detecting the electron density fluctuation of the carbon material after hydrogen saturation.

## 1. Introduction

Due to conventional energy source depletion and serious environmental pollution conditions, a large number of investigations have been devoted to the problem of hydrogen use in energetics due to its high-efficiency and environmental friendliness. The unsolved problems of hydrogen storage and transportation hinder large-scale use of hydrogen. Currently, existing storage methods which presuppose adsorption at low temperatures, under high pressure, in the form of metal hydrides and intermetallic compounds, or in the liquid state are not efficient enough for providing safety of reversible storage and compacted transportation of hydrogen [1,2]. We must investigate the influence of the saturation parameters on material sorption capacity when considering the storage of hydrogen during its transportation. The use of ultra-high or ultra-low temperature and pressure leads to further constructional changes during hydrogen transportation. The main requirement for hydrogen accumulator materials is a gas content with a weight less than 6.5% [3].

The application of carbon nanomaterials as an accumulator is one of the promising methods for hydrogen storage. Nanodiamonds have a unique structure that allows the use of the material as an adsorbent. There have been a large number of works devoted to the study of the sorption properties of carbon materials published in recent decades [3,4,5,6,7,8].

The hydrogen sorption capacity in nanodiamonds reaches outliers. In addition, typically, nanodiamonds are treated with hydrogen to restore the surface. This process is only an intermediate one for further modification of nanodiamonds [9]. In the case of a defect-free nanodiamond core, hydrogen treatment only changes the surface. This is related to the large number of free bonds on the surface. The presence of defects in a diamond core should also have an impact on hydrogen sorption.

Positron annihilation (PA) techniques, such as positron lifetime spectroscopy (PLS) and Doppler broadening spectroscopy (DBS), are promising nuclear-physical methods for investigating structural defects in different materials, including ultrafine-grained and nanostructured materials [10,11,12,13]. The efficiency of PA methods for studying carbon based materials was demonstrated in [14,15]. These methods allow the solution to such problems as the study of the mechanisms and dynamics of the occurrence, transformation, and disappearance of defects during hydrogenation. In particular, PLS enables to determine the type of defects, a perform tracking of the dynamics of their concentration and size during hydrogenation. DBS can be used to investigate structural changes, phase transitions, and chemical composition.

Furthermore, positronium (Ps) can be used as a sensor for detecting places with the most effective capture of positively-charged particles, including hydrogen atoms. At present, not many works describe the application of PA methods to study carbon nanomaterials’ porous structure. However, experimental and well-described theoretical research, dedicated to porosimetry by PA on metals and alloys, has taken place [16,17,18,19,20]. Therefore, the aim of this work was to investigate the changes in the positron characteristics of nanodiamonds before and after cyclic hydrogen saturation.

## 2. Experimental Methods

### 2.1. Materials

**Figure 1:**
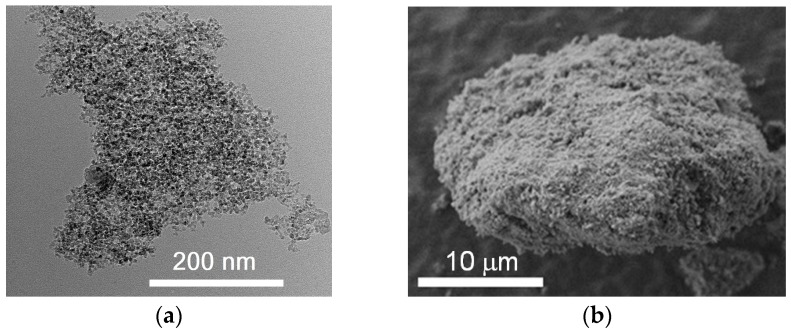
Transmission (**a**) and scanning (**b**) microscopy micrographs of nanodiamonds.

The carbon-based material (Figure 1) was formed by detonation synthesis. Synthesis of nanodiamonds was carried out by detonation of solid explosives in an inert atmosphere. The release of very large capacities of energy occurred due to the breaking of chemical bonds in the front of the detonation wave. The highly-dispersed carbon material was condensed from the liberated carbon under conditions of high temperatures 3000–4000 K and pressures 20–30 GPa. Unpurified material contained 25.0–50.0 wt % of nanodiamonds. The medium purity material was chosen for the studies. The purified material consisted of nanodiamonds, onion-like particles, and a certain amount of amorphous carbon. The material was purified by the Federal Research and Production Center “Altai” (Biysk, Russia). The nanodiamonds consist of a crystalline diamond core and a non-diamond shell with functional groups on the surface.

### 2.2. Sample Preparation

Compression is a type of powder compaction which is important when studying material sorption characteristics. The weight, volume, and density of a pill can be measured more accurately due to compacting. The carbon material was compacted by a hydraulic press at a pressure of 400–620 MPa. The weight of each sample was 0.1 g.

### 2.3. Characterization

X-ray diffraction (XRD) is the most common investigation method to study carbon–based materials. Diffraction is the non-destructive testing method that provides statistical evaluation of characteristics of a macroscopic sample. X-ray diffraction measurements were carried out using Shimadzu XRD-7000 high-resolution powder diffractometer (Shimadzu Corporation, Kyoto, Japan) with CuKα-radiation.

The structure of the nanodiamonds was investigated by transmission electron microscopy (TEM). Nanodiamond morphology was examined by a JEM-2100F electron microscope (JEOL, Tokyo, Japan). The limiting resolution (lattice) was 0.1 nm. The limiting resolution (by points) was 0.23 nm. The accelerating voltage was 200 kV. The diameter of the spot was 2–5 nm. The maximum magnification was 1,500,000 times.

Raman spectroscopy provided information about the purity, types of carbon allotropes, and structure imperfections. The method was abundantly used for identifying the carbon structures, as well as their defectiveness.

Raman spectroscopy is sensitive to the orientation of the carbon-carbon bond changes. Typically, the spectra of carbon allotropes are similar in appearance. The presence of a D-reflex at 1332 cm^−1^ corresponded to the diamond structure or carbon in sp^3^-state. The presence of G-reflexes in the region of 1580 cm^−1^ corresponded to graphite or carbon in the sp^2^-state. The presence of bands at 500 cm^−1^ indicated the presence of amorphous carbon in the material. Additional bands in the spectrum indicated the presence of various carbon-carbon bonds in the structure of graphite. The change of defectiveness of the material was estimated by the ratio of the intensities of D and G bands. Nanodiamond structural changes were registered by a Centaur U HR complex with a Raman spectrometer with the use of laser excitation at a wavelength of 532 nm (Spectrum Instruments Ltd., Limerick, Ireland).

### 2.4. Hydrogenation Studies

Hydrogen concentration was measured by gas reaction controller complex (Advanced Materials Corporation, Pittsburgh, PA, USA) [21,22] at low temperature and a pressure of 0.6 MPa. The choice of pressure was due to the limitation of the hydrogen generator. The samples were placed in a chamber, which was then evacuated to a pressure of 10^−7^ MPa. The process of hydrogen inflowing occurred after evacuation. The concentration-time sorption isotherms are presented below. The main information of the isotherms is the concentration of hydrogen absorbed by nanodiamonds.

### 2.5. Positron-Annihilation Studies

Investigation of positron lifetime (PL) and the Doppler broadening shift (DB) of the annihilation line before and after hydrogenation was performed using the special complex. The samples were arranged in a so-called “sandwich” and mounted in a special sample holder. PL and DB spectra were collected simultaneously on positron spectrometer (Tomsk Polytechnic University, Tomsk, Russia). The positron source was represented by the ^44^Ti isotope with an activity of 24.5 μCi. The average depth of positron penetration was determined by the method proposed in [23] and it is equal to ~650 μm. Three PL spectra with 4 × 10^6^ counts each were collected for every sample. Spectra were fitted using LT10 software (University of Silesia, Katowice, Poland).

**Figure 2:**
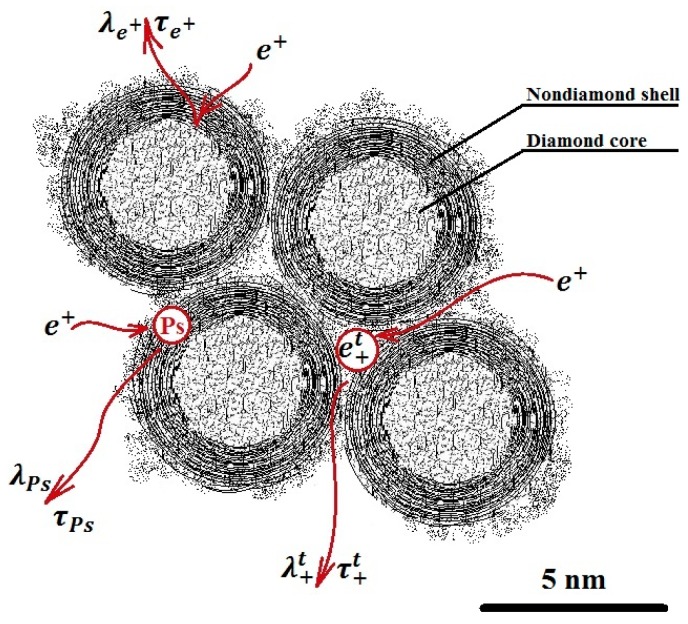
Model of delayed formation of positronium in nanodiamonds.

The spectral analysis was performed by implementing the delayed formation of the positronium (DFP) model, as illustrated in Figure 2. The choice of the DFP model was due to the positronium sensitivity to the pores’ structure and free volumes, as well as the electron density at the site of its localization.

The DFP model assumes two channels of positronium (Ps) formation. There is the quick channel, in the blob, and a slower channel, where an unbound positron that has left the blob diffuses through the material and annihilates with the rate λ_+_, or it is captured by some trapping center and then it annihilates with the rate λ^t^_+_, or it forms a Ps with a shallow trapped electron [24]. Several parameters, such as *τ*_o-Ps_—o-Ps pick-off lifetimes, *τ*_T_—lifetime of trapped positrons, *τ*_free_—lifetime of free positrons, *I*_o-P_—intensity of o-Ps pick-off component, *I*_T_—intensity of trapped positrons component, *μ*—positron trapping rate to positron traps, and *κ*—rate of Ps formation from a positron and a trapped electron, were used for analysis after the background and the source correction.

**Figure 3:**
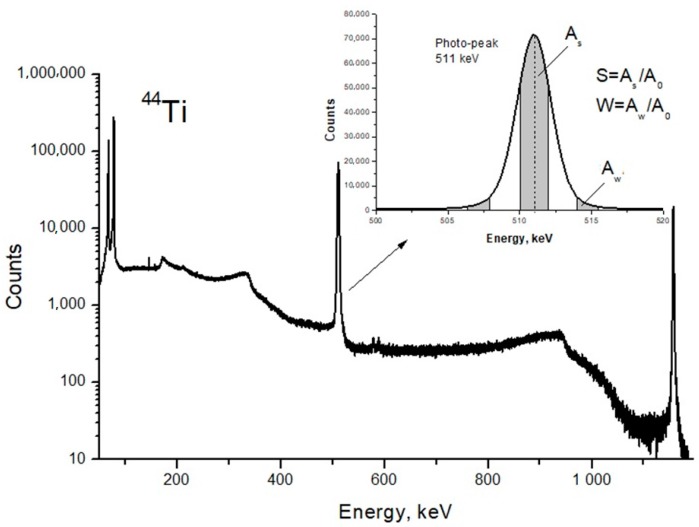
Energy spectrum of scintillator BaF_2_ detector and line shape parameters of the photo-peak.

DB spectra were acquired by collecting 2.5 × 10^5^ counts and analyzed using the SP software package (Institute of Nuclear Physics PAS, Krakow, Poland). The line-shape of *S*- and *W*-parameters were also evaluated using the aforementioned software [25]. The *S*-parameter is the ratio of the central peak area to the entire area of the Gaussian distribution; the *W*-parameter is the ratio of the Gaussian distribution side area to the entire area (Figure 3). The *S*-parameter is sensitive to the annihilation with low momentum valence and unbound electrons. The *W*-parameter depends on high-energy core electrons.

Both the *S*- and *W*-parameters depend on the concentration and type of defects [25]. The authors of work [26] suggest the parameter *R* = *S/W* that does not depend on the concentration of defects and is determined only by their type. The graphical method for analyzing the parameters of the DBS shape was used. According to [27], if the experimental values of the *S*- and *W*-parameters for the set of samples are in the straight line of the plot *S* = *f*(*W*), this means that the predominant positron traps in the samples are similar types of defects and the *R*-parameter is determined by the inclination of the straight line. Thus, the change of the straight line (*S*, *W* of the plot *S* = *f*(*W*)) represents the change of the *R*-parameter and predominant positron trapping state.

## 3. Results and Discussions

### 3.1. Sample Preparation

**Figure 4:**
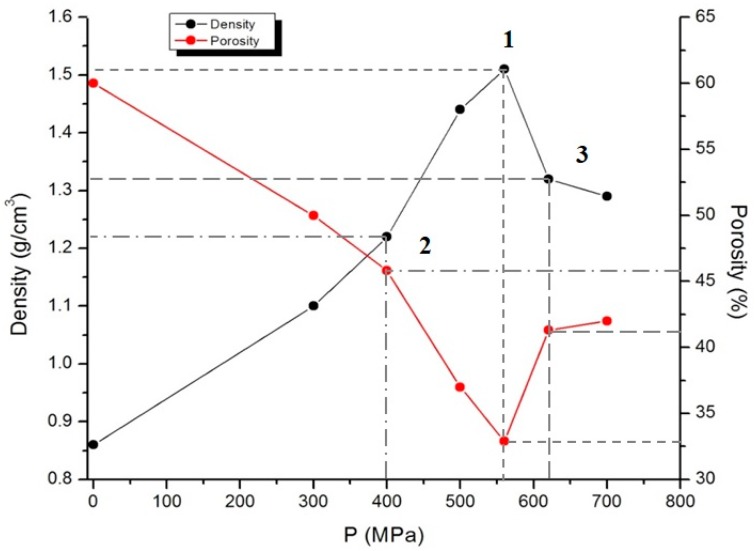
Density and porosity-pressure diagram for samples 1–3.

The compacted carbon powders were used for hydrogen saturation and positron annihilation measurements. The compaction process was carried out on hydraulic press at the room temperature in the pressure range of 100–700 MPa without using a binder. Under the pressure below 400 MPa, the material stays uncompacted, while under pressure above 620 MPa, it destructs due to high internal stresses. The samples are pills with 5 mm diameter and mass of 0.1 g. The density of the sample was calculated through the ratio of the mass of the compacted sample to its volume. The densities of samples 1, 2, and 3 were 1.51, 1.22, and 1.32 g/cm^3^ and porosity was 32.9, 45.8, and 41.3%, respectively (Figure 4). Porosity was calculated by the formula:

$$P=\left(1-\frac{\rho_{v}}{\rho_{t}}\right)\times 100\%,$$

where $\rho_{v}$ is the volume density, and $\;\rho_{t}$ is the true density.

### 3.2. Structure Investigation

**Figure 5:**
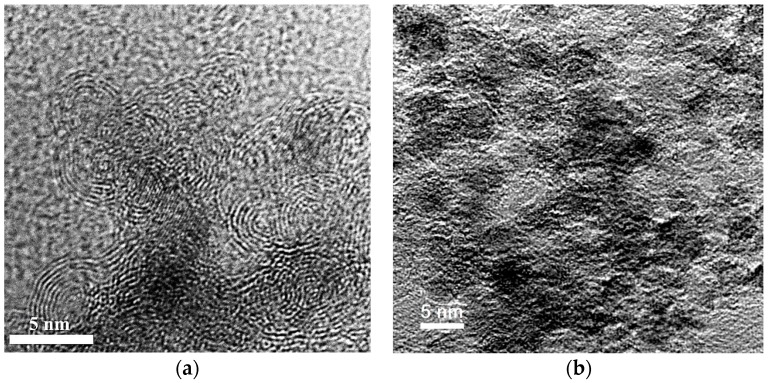
TEM images of the nanodiamonds material: (**a**) carbon onions; and (**b**) nanodiamonds.

According to the data obtained by transmission electron microscopy it can be argued that nanodiamonds are present in the material in a different state. In addition to the standard structure with the diamond core covered with a non-diamond shell, the particles with bulbous shape are observed. Further, it can be assumed that all the diamond particles are in some disordered shell. As further methods have not revealed the presence of amorphous carbon, it can be assumed that the structure is similar to graphite in the sp^2^-state. Figure 5 shows a carbon material containing nanodiamonds with particle sizes below 10 nm.

### 3.3. Hydrogen Storage Capacity

**Figure 6:**
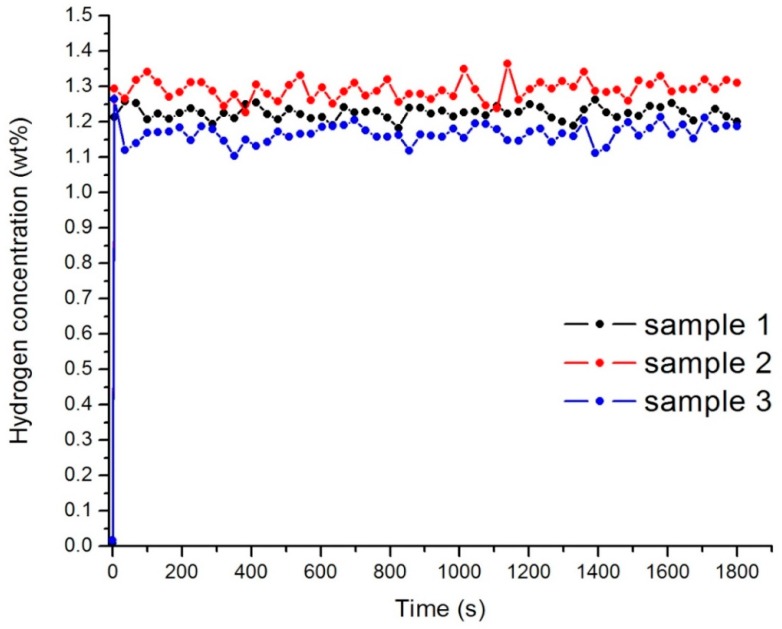
Hydrogen concentration in the samples 1–3 after the first cycle of sorption.

The samples were hydrogen saturated at the low temperature of 243 K and the pressure of the hydrogen was 0.6 MPa. The accumulation of hydrogen by the material is due to physical molecular sorption. For samples 1–3 the capacity of hydrogen was near 1.2 wt % at 243 K after the first cycle of hydrogenation (Figure 6). Hydrogen concentration does not change significantly with the increase of the cycles of sorption. This value of capacity can be explained by the inability of hydrogen to penetrate into the core of the nanodiamond.

**Figure 7:**
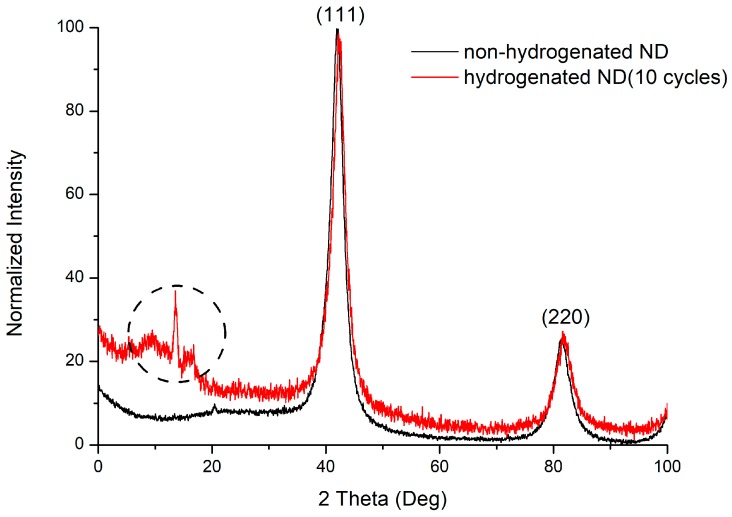
Diffraction pattern of non-hydrogenated and hydrogenated nanodiamonds.

The reflexes on the diffraction pattern (Figure 7) correspond to the structure of a cubic diamond. The reflections (111) and (220) are present on the diffraction pattern. The allocated range is not identified; however, it can be associated with the three-component compounds formation (C–H–N). The presence of nitrogen in the material is due to the synthesis and treatment processes. The charges of trinitrotoluene with hexogen in the nitrogen atmosphere are usually used for carrying out the detonation synthesis of ultradisperse diamonds. The surface of the nanodiamond is usually contaminated with nitrogen impurities due to purification with nitric acid. The dimensions of the coherent scattering regions for nanodiamond samples are, on average, 4 nm.

**Figure 8:**
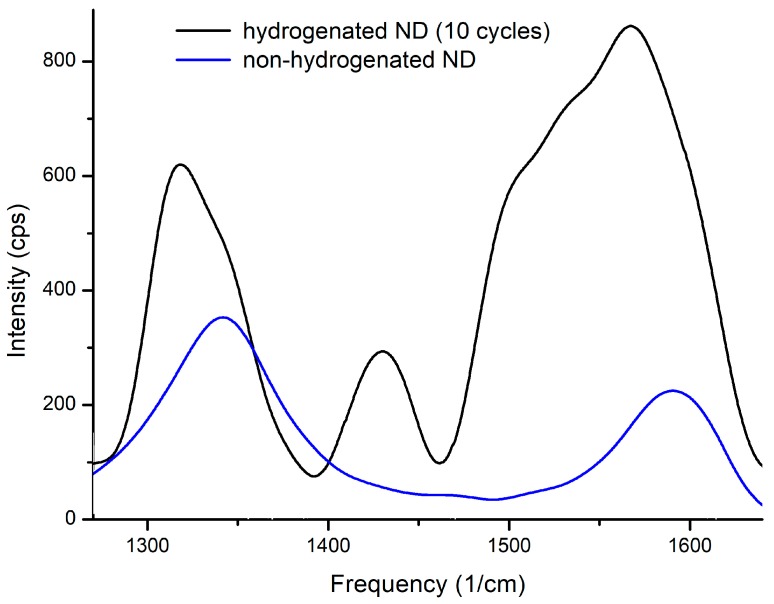
Raman spectroscopy of non-hydrogenated and hydrogenated nanodiamonds.

Raman spectra of nanodiamonds before and after hydrogenation are presented at Figure 8.

The spectra of the three samples before hydrogen saturation are identical (blue line). As mentioned above, the presence of D and G peaks indicates the presence of carbon in the sp^2^- and sp^3^-states. It was also observed that the ratio of the intensities of these reflexes varies after hydrogen sorption-desorption cycles. The intensity of G-band increases indicating the increase in carbon in the sp^2^-state. After hydrogenating the peak assigned to the CH deformation modes (1440–1450 cm^−1^) is clearly discernible (see Figure 8). The intensity ratio I_D_/I_G_ is presented in Table 1.

The main causes of additional bands and the reduction of the frequency of the fundamental diamond bands are the numerous lattice distortions at the edge of the crystal. The band at 1600 cm^−1^ is a low-frequency band and the shoulder bands D indicate the presence of carbon in the sp^2^-state due to surface defects.

### 3.4. Positron Spectroscopy

**Figure 9:**
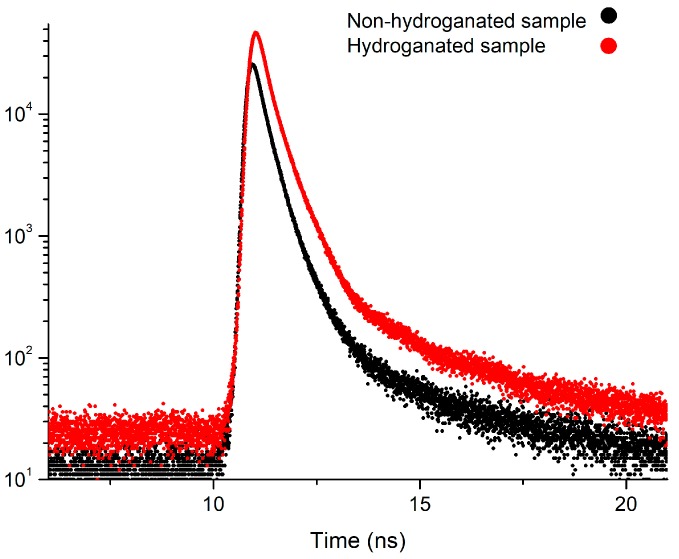
Positron annihilation lifetime spectra measured for non-hydrogenated and hydrogenated nanodiamond samples.

**Figure 10:**
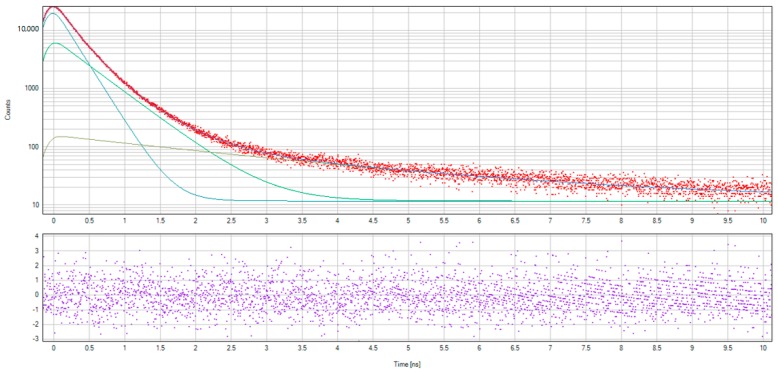
The examples of the fitting spectra using LT10 software.

The spectra of non-hydrogenated and hydrogenated samples of nanodiamonds and examples of fitting spectra using LT10 software are presented in Figure 9 and Figure 10.

The positron lifetime components and intensity for nanodiamond samples before and after hydrogenation are presented in Table 2.

The positron annihilation studies of nanodiamond samples before hydrogenation reported three components in the positron lifetime spectra indicating positron trapping (*τ*_T_), ortho-positronium formation (*τ*_o-Ps_), and free positron annihilation (*τ*_free_). The samples before hydrogenation are practically identical. The component *τ*_free_ averaged 0.31 ± 0.04 ns with the greatest intensity 82%. The value of this component is slightly lower than of the component reported by other authors in nuclear graphite samples, i.e., 0.36–0.38 ns [28]. Our estimation of positron lifetime in the graphite crucible (LECO) after degasification shows two components, *τ*_free_ = 0.28 ± 0.02 ns and *τ*_T_ = 0.42 ± 0.01 ns. The short lifetime observed for graphite has been ascribed to free positrons and the longer lifetime has been ascribed to surface-trapped positrons [29,30]. This fact confirms our assumptions that *τ*_free_ is related with the annihilation of free positrons in a graphite-like structure. Small fluctuations of the value of this lifetime component after hydrogen exposure do not change this interpretation.

Annihilation from the positron trapping state is carried out during the lifetime *τ*_T_ (0.52 ± 0.02) ns with intensity 13.9 ± 0.7%. This component could be related with the annihilation of positrons trapped on the outer surface of the nanodiamonds [31]. The component *τ*_o-Ps_ is related with formation and annihilation of o-Ps at the free volume and averaged 3.12 ± 0.05 ns for samples 1–3. The intensity of the component ranged from 2.9% to 3.4%. Taking into account the Tao–Eldrup model [32,33] we can estimate the radius of the pore to be equal to ~0.252 nm for the case of the non-hydrogenated samples. In addition, since the possibility of the formation of positronium in diamonds is still being discussed [34], we can assume that the formation of positronium is due to the large number of adsorbed gases and moisture because the measurements were carried out in air under normal conditions. For both samples (2,3) after the first loading of the hydrogen, the value of the *τ*_o-Ps_ component was increased to the value of ~4.2 ns, however, its intensity practically did not change. The significant change was observed after the tenth hydrogenation. The value of lifetime was reduced to 2.35 ns and the intensity increased to the value of ~5% after the tenth cycle.

It should be noted that there is no component with lifetime 100–110 ps associated with the annihilation of positrons in the bulk of diamond [34,35,36]. For defect-free diamonds, the diffusion length is *L*_diff_ = 162 nm [34]. Based on the fact that the average size of the nanodiamond is less than 10 nm (Figure 4) the probability of positron annihilation from this state is negligible.

The short-lived component *τ*_free_ and intensity *I*_free_ increased after the first cycle of hydrogen saturation and decreased after the tenth cycle to 0.29 ± 0.02 ns and 53%, respectively, which is related with the change in the bulk of samples. The middle-lived component *τ*_T_ increased after the first cycle of hydrogen saturation and decreased to 0.42 ± 0.01 ns after the tenth cycle of sorption and it was similar as for non-hydrogenated graphite, however, the intensity I_T_ decreased to 7.4–10.3% after the first cycle of sorption, and increased to 42.3% after the tenth cycle.

The results obtained by DBS are in good agreement with the results presented in Figure 8.

**Figure 11:**
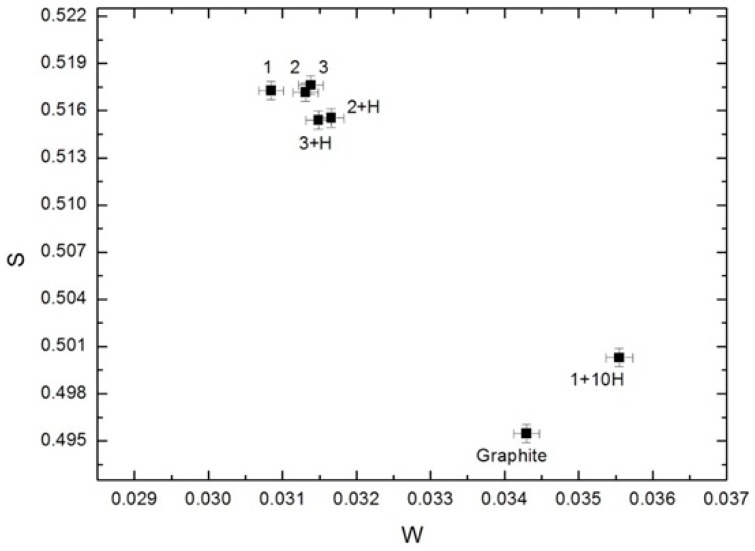
Dependence of the *S*-parameter on the *W*-parameter for non-hydrogenated and hydrogenated nanodiamond samples.

From Figure 11 we can see that *S*- and *W*-parameters for samples before hydrogenation are almost identical. The first cycle of hydrogen sorption slightly reduces the *S*-parameter, but the *W*-parameter does not change. The inverse changes for *S*- and *W*-parameters were observed in the tenth cycle. The indicated changes completely coincide with the changes of *τ*_T_ depending on the hydrogenation cycles. Thus, the DBS data mainly characterize the annihilation of positrons trapped on the outer surface of nanodiamonds.

## 4. Conclusions

Nanodiamond samples with different density and porosity were prepared by hydraulic pressing. The hydrogen concentration of the samples at the temperature of 300 K and the pressure of 0.6 MPa was 1.2 wt %. The porosity of the carbon material before hydrogenation revealed no considerable impact on the positron lifetime and Doppler broadening. In spite of the fully-desorbed hydrogen, the positron annihilation parameters changed. There is no component with a lifetime of 100–110 ps associated with the annihilation of positrons in the bulk of diamond, therefore, we assumed that positrons annihilate in the disordered surface area of nanodiamond. The nanodiamonds’ hydrogen saturation leads to the change of the lifetime of trapped positrons and ortho-positronium, intensity, and *S*- and *W*-parameters.

Consequently, these results demonstrate that positron spectroscopy can be used to study carbon hydrogen storage materials.

## Figures and Tables

**Table 1:** The intensity of D and G peaks.

Sample	*I*_D_	*I*_G_	*I*_D_/*I*_G_
0 cycles	352	213	1.65
1 (10 cycles)	623	891	0.70

**Table 2:** The parameters of positron annihilation in the nanodiamonds before and after hydrogenation.

State	*τ*_o-Ps_, ns	*I*_o-Ps_, %	*τ*_T_, ns	*I*_T_, %	*μ*, ns^−1^	*τ*_free_, ns	*κ*, ns^−1^
1	3.05 ± 0.04	3.35	0.51 ± 0.01	14.8	0.60 ± 0.02	0.30 ± 0.03	0.18 ± 0.01
2	3.13 ± 0.06	2.9	0.52 ± 0.01	13.6	0.53 ± 0.02	0.31 ± 0.04	0.16 ± 0.01
3	3.18 ± 0.06	3.28	0.53 ± 0.02	13.2	0.51 ± 0.02	0.31 ± 0.04	0.17 ± 0.01
2 (1 cycle)	4.47 ± 0.08	2.82	0.59 ± 0.01	10.3	0.37 ± 0.01	0.32 ± 0.04	0.14 ± 0.01
3 (1 cycle)	4.1 ± 0.1	3.19	0.69 ± 0.09	7.37	0.24 ± 0.07	0.34 ± 0.04	0.14 ± 0.01
1(10 cycles)	2.35 ± 0.04	5.07	0.42 ± 0.01	42.3	2.91 ± 0.15	0.29 ± 0.02	0.46 ± 0.01
Graphite	-	-	0.42 ± 0.01	23.10	2.7 ± 0.1	0.28 ± 0.02	-
